# Development and Evaluation of a Novel Relatively Low-Cost Method to Derive HIV-1 Integration Sites and Proviral Sequences

**DOI:** 10.3390/v18030311

**Published:** 2026-03-02

**Authors:** Samantha R. Hardy, Sheila Styrchak, Tim De Meyer, Laurens Lambrechts, Tine Struyve, Basiel Cole, Liesbet Termote, Sherry McLaughlin, James I. Mullins, Linos Vandekerckhove, Lisa M. Frenkel

**Affiliations:** 1 Center for Global Infectious Disease Research, Seattle Children’s Research Institute, University of Washington, Seattle, WA 98109, USA; 2 Department of Data Analysis and Mathematical Modelling, Ghent University, 9000 Ghent, Belgium; 3 HIV Cure Research Center, Department of Internal Medicine and Pediatrics, Ghent University Hospital, Ghent University, 9000 Ghent, Belgium; 4 Department of Microbiology, University of Washington, Seattle, WA 98109, USA; 5 Departments of Pediatrics and Laboratory Medicine and Pathology, University of Washington, Seattle, WA 98101, USA

**Keywords:** multiple displacement amplification (MDA), integration site (IS), provirus, HIV-1, whole-genome amplification (WGA), REPLI-g MDA

## Abstract

In people taking antiretroviral therapy (ART) for HIV infection, the methods to characterize latent and active HIV reservoirs remain costly and labor-intensive. Our objective was to develop a relatively low-cost technique to amplify and sequence the proviruses that persist during ART along with the site in the human genome where each provirus is integrated. We developed a novel HIV-specific Multiple Displacement Amplification (**HIV-MDA**) assay that specifically amplifies HIV-1 proviruses and their associated integration site. Upon comparison of our HIV-MDA to an established commercial kit designed to amplify cellular DNA, we found that the HIV-MDA (1) typically yielded a greater number of HIV integration site (**HIV IS**) sequences per 150,000 cells analyzed; (2) improved rates of proviral DNA amplification; and (3) amplified HIV IS at a fraction of the cost (13.6 times less expensive). Thus, the HIV-MDA method appears to be a more sensitive and cost-effective approach to sequencing HIV IS and the associated proviruses compared to a commercial kit.

## 1. Introduction

Presently, the barriers to HIV-1 cure are reservoirs of infected cells with replication-competent proviruses that persist despite long-term antiretroviral therapy (**ART**) and rebound following cessation of ART [1,2,3,4,5]. To extinguish HIV replication without ART, strategies are needed to target and eliminate viral reservoirs that persist during ART [2,6]. These reservoirs are maintained in part by the clonal expansion of infected CD4+ T cells, which are typically identified by recovering identical integration sites [7,8,9,10,11]. Sequencing of the proviruses that persist during ART and their linked viral integration sites is one method used to characterize the reservoir to be targeted to “cure” the infection. Multiple approaches for sequencing both proviruses and viral integration sites have been published: Matched Integration Site and Proviral sequencing (**MIP-seq**) [9], Simultaneous TCR, Integration site and Provirus sequencing (**STIP-Seq**) [12], Multiple Displacement Amplification–Single Genome Sequencing (**MDA-SGS**) [13], Parallel HIV-1 RNA, integration site, and Proviral sequencing (**PRIP-seq**) [14], and the Individual Proviral Sequencing Assay (**IPSA**) [15]. All of these assays include a step that employs Multiple Displacement Amplification (**MDA**) for whole-genome amplification (**WGA**) of human DNA containing a single proviral genome. Depending on the assay, this is achieved either by limiting dilution of genomic DNA or single-cell sorting of HIV-positive cells, typically using a pre-assembled whole-genome amplification kit. These approaches, even if limited to negatively selected CD4+ cells, incur a high cost to sequence relatively few intact HIV proviral sequences and the associated HIV integration site (**HIV IS**) due primarily to the rarity of intact proviruses, which may comprise only 0.5–5% of the persisting HIV DNA [16,17,18], coupled with the relatively high cost of reagents [19]. We developed a relatively low-cost HIV-MDA with targeted amplification of the HIV integration site (**HIV IS**) and the linked proviral genome from single-infected cells. We compared our novel HIV-specific Multiple Displacement Amplification (**HIV-MDA**) method to one that uses a WGA kit, REPLI-g Single Cell Kit (cat. #150345, QIAGEN, Hilden, Germany), here referred to as REPLI-g MDA. Our data show similar or greater efficiencies in the detection of HIV IS and sequencing of proviral genomes at a fraction of the cost.

## 2. Materials and Methods

### 2.1. Specimens/Cohort

Cryopreserved peripheral blood mononuclear cells (**PBMC**) were obtained by leukapheresis from individuals in two cohorts, the HIV Sequencing After Treatment Interruption to Identify the Clinically Relevant Anatomical Reservoir (**HIV-STAR**) [20] and the Seattle Primary Infection Cohort (**PIC**) [21]. Participants were adult males with documented HIV-1 subtype B infection who were receiving suppressive ART. Selection criteria included sustained ART-mediated suppression of plasma HIV-1 RNA (<40 copies/mL) for greater than 6 months prior to leukapheresis and the availability of multiple PBMC aliquots from a single leukapheresis.

### 2.2. Specimen Processing and HIV Quantification

CD4+ cells were purified by negative selection from PBMC using the EasySep Human CD4+ T cell isolation kit (STEMCELL Technologies, Vancouver, British Columbia, Canada), except for single participants for whom bulk PBMC or sorted CD4+ cells were assayed. DNA was extracted from cells using the Wizard Genomic DNA purification kit (cat. #A1120, Promega, Madison, WI, USA). HIV DNA load was quantified by qPCR amplification of the HIV 5′ LTR [22] or via a viral ORF detection assay (**VODA**), a multiplex qPCR targeting HIV LTR, env, gag regions and human transferrin receptor (**hTFR**) [23].

### 2.3. HIV-MDA Workflow

**Figure 1:**
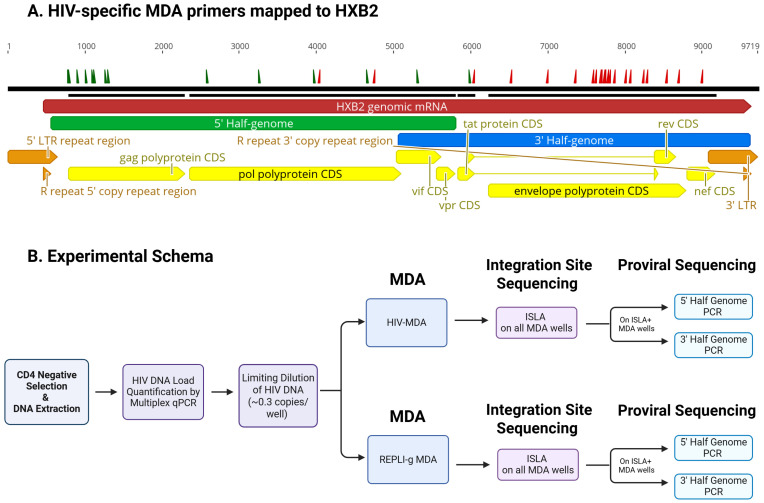
**Experimental Schema to compare the HIV-specific multiple displacement amplification (HIV-MDA) to the REPLI-g MDA.** (**A**) HIV-MDA primer pools mapped to HIV-1 HXB2 reference genome. The 15 forward primers (green tick marks along the top) and 26 reverse primers (red tick marks; sequences in Table S1) were designed to amplify both intact and defective HIV proviruses using a 2-amplicon half-genome approach. Primers annealing within the 5′ region of the provirus generate amplification products extending toward the 3′ end (blue bar), whereas primers annealing within the 3′ region generate products extending toward the 5′ end (green bar). Image generated using Geneious Prime 2021.0.3 (Biomatters, Inc., Auckland, New Zealand). (**B**) Experimental workflow used to compare HIV-MDA to REPLI-g MDA. MDA products were subjected to integration site loop amplification (ISLA) to identify viral integration sites. ISLA-positive MDA wells were subsequently subjected to proviral 5′ and 3′ half-genome PCR. (Created in BioRender. Hardy, S. https://BioRender.com/numwhzk (accessed on 16 February 2026)).

HIV-MDA was conducted using two pools of primers positioned as shown (Figure 1A); one pool contained 15 forward primers designed to anneal to the 5′ end of the negative strand of the HIV-1 provirus and the other pool contained 26 reverse primers that were designed to anneal to the 3′ end of the positive strand of the provirus (Table S1). Primers were designed using alignments of HIV-1 subtype B sequences randomly selected from U.S.-based infections in the Los Alamos HIV database, with searches restricted to one sequence per subject to minimize sampling bias. To improve alignment quality, the genome was divided into four regions and aligned separately using at least 100 sequences per region (HXB2: 0–626, 173 sequences; HXB2: 626–3626, 118 sequences; HXB2: 3600–6626, 354 sequences; HXB2: 6600–9026; 487 sequences). All primers contain phosphorothioate bonds at the ultimate and penultimate 3′ positions to promote strand displacement and resist exonuclease degradation. Equimolar pools of primers were diluted to a total concentration of 400 µM. Primer concentrations were optimized using ACH2 cells (obtained through the NIH HIV Reagent Program, Division of AIDS, NIAID, NIH, contributed by Dr. Thomas Folk) to amplify across HIV-1 LTR, gag, and env regions.

HIV genomes were quantified in extracted DNA by qPCR and diluted prior to MDA to achieve limiting dilution conditions, such that approximately 30% of MDA reactions were expected to be positive. This provides an approximately 80–85% likelihood that most positive wells contain a single proviral genome, consistent with a Poisson distribution, minimizing the likelihood of co-amplification of multiple proviruses (Figure 1B). The HIV-MDA was carried out following an established MDA-SGS method [13], in which multiple displacement amplification is performed using phi29 DNA polymerase under isothermal amplification conditions to generate long DNA fragments. In this approach, random hexamer primers initiate strand-displacement synthesis, allowing for unbiased whole-genome amplification. Two modifications were introduced to the MDA step: HIV-specific MDA primers (10 µM/well) were added to enhance proviral enrichment, and the concentration of random hexamer primers was reduced from 50 µM/well to 25 µM/well to limit nonspecific background amplification.

### 2.4. REPLI-g MDA

To conduct the REPLI-g MDA, DNA was amplified using the REPLI-g Single Cell kit, which uses only random hexamer primers. These reactions were conducted according to the manufacturer’s instructions, with two modifications: the volume was scaled down to a 40 µL final volume (to reduce the cost/reaction) and the MDA incubation was shortened from 8 to 4 h per modified MIP-seq protocol (K. Einkauf, personal communication, 2020).

### 2.5. HIV Integration Site Capture—ISLA

To detect HIV IS from both HIV-MDA and REPLI-g MDA reactions, MDA products (10 µL/reaction) were subjected to integration site looping amplification (ISLA) [7], a nested PCR-based approach that selectively amplifies host–virus junctions to identify HIV integration sites. ISLA reaction products were visualized on 1% agarose gels, with positive reactions showing a band between 500 and 2500 bp. Gel-positive reactions were sequenced (Sanger) and processed using a computational pipeline to identify the HIV IS (https://integrationsites.fredhutch.org/).

### 2.6. HIV Proviral Amplification and Library Preparation

Proviral amplification was performed using MDA products from wells that yielded an integration site from ISLA (Figure 1B). To increase the sensitivity of amplifying near full-length proviral genomes (NFLG, HBX2 positions 581–9605 bp) at a moderate cost we targeted two overlapping regions, each comprising approximately half of the HIV genome (Figure 1A). The 5′ and 3′ half genomes were amplified in separate 25 µL nested PCR reactions using 1 µL of MDA product (primers listed in Table S2).

For half-genome amplification, the 25 µL first-round PCR mix consisted of 5 µL of 5× Prime STAR GXL buffer, 2 µL of 2.5 mM dNTP mix, 0.5 µL of PrimeStar GXL polymerase (Takara Bio, Kusatsu, Shiga Prefecture, Japan #R050B), 0.125 µL of ThermaStop #TSTOP-500 (Sigma Aldrich, St. Louis, MO, USA), 250 nM of forward and reverse primers, and 1 µL of MDA product. The second-round PCR was composed of the same reaction mix with 1 µL of the first-round product as input. Thermocycling conditions for both PCR rounds were as follows: 2 min at 98 °C; 35 cycles (10 s at 98 °C, 15 s at 62 °C, and 5 min at 68 °C); and 7 min at 68 °C. To improve detection of frequently deleted proviruses, two alternative forward primer sets (Table S2) were used for amplification of the 5′ half genome.

To further evaluate whether amplification was precluded by proviral deletions or mutations where the half-genome primers anneal, NFLG amplification was performed on a subset of samples using two different forward primer sets (Table S2) encompassing HXB2 positions: 554–9665 and 638–9665. The composition of the PCR reaction mix was the same as described for half-genome amplification. First-round thermocycling conditions were as follows: 2 min at 98 °C; 35 cycles (10 s at 98 °C, 15 s at 62 °C, and 7.5 min at 68 °C); and 7 min at 68 °C. The second-round PCR used the same reaction mix with input of 1 µL of the first-round product. Second-round thermocycling conditions were as follows: 2 min at 98 °C; 25 cycles (10 s at 98 °C, 15 s at 64 °C, and 5 min at 68 °C); and 7 min at 68 °C.

PCR products were assessed on 1% agarose gels. Amplicons of 750–9100 bp length were tagged with unique index adaptors and prepared for long-read sequencing using the PacBio SMRTbell prep kit 3.0 (PacBio, Menlo Park, CA, USA) [24].

### 2.7. Proviral Sequencing and Analysis

Sequencing was performed using the Pacific Biosciences Sequel II instrument (Menlo Park, CA, USA) followed by demultiplexing and processing into consensus sequences for each sample using the SGA pipeline (https://github.com/MullinsLab/sga_index_consensus accessed on 16 February 2026). Pipeline-generated outputs were used to assess proviral sequence integrity and overall quality. Half-genome sequences derived from the same MDA well were aligned using Geneious Prime 2021.0.3 (Biomatters, Auckland, New Zealand) and assembled into NFLG when the overlapping regions (HXB2: 5088–5783) were identical within a single nucleotide. Sequences generated in this study have been deposited in GenBank with accession codes PX940098–PX940529.

### 2.8. Statistical Tests

#### 2.8.1. Integration Site Analyses

The yields of HIV integration sites by the two MDA methods were compared using a paired *t*-test. To evaluate sampling of the populations of HIV IS, rarefaction curves were generated by random sampling of a defined number of HIV IS and plotting the proportion of unique IS. The R vegan package (version 2.7-2) [25] was used, with step size 1 and—for the method with most IS detected—random subsampling with a size equal to the lower number of IS detected for the other method. The slopes were visually assessed for saturation and compared across participants.

#### 2.8.2. Integration Site Clonality

To evaluate quantitative reproducibility of the MDA methods, the number of clonal HIV IS detected were compared between both assays by a linear model. As this analysis requires the clear presence of clonality, it was restricted to the two participants with the most clonal IS (STAR10 and STAR11). First, we defined clonal IS as those IS that were detected at least twice in the participant, independent of the MDA method (e.g., twice by one method or once by both methods). Subsequently, linear regression was used to compare the fraction of IS per clone detected between both methods and deviation from perfect correspondence (direction coefficient = 1) was evaluated.

Additionally, we combined the quantitative measurements of clonal IS for both methods over all samples in a single figure. Note that this is merely for visualization purposes, as major differences between samples (degree of clonality) and depth of analysis (total numbers of IS detected, most often lower for the REPLI-g MDA results) impeded formal statistical analysis. To indicate the impact of the number of IS analyzed per sample, the size of the plotted points was linearly rescaled as a function of the square root of the minimal number of IS measured (by one of the MDA methods) for that sample (“Number of IS”, Table 1).

#### 2.8.3. Overdispersion Analysis—Clonal Integration Site Detection

Since both MDA methods most likely detected only a small fraction of the total number of IS present in each person, the presence of random sampling effects may provide an explanation for observed differences between the methods. If that is the case, sequencing of increasingly more IS per sample for each method will lead to increasing quantitative correspondence between both methods. To evaluate whether random sampling may indeed be sufficient to explain observed quantitative differences between the methods, we again focused on the counts for clonal IS. Under random sampling, the standard deviation between the methods’ counts for a clonal IS should be a binomial distribution, with the prior chance of detection by a method proportional to the total number of clonal IS detected by that method. If the methods’ counts differ more than can be explained by random sampling, the standard deviation between the method’s counts will be larger, i.e., there will be overdispersion. To evaluate overdispersion, a paired *t*-test was used to compare the observed standard deviations between both methods’ counts with the expected standard deviations for all clonal integration sites combined. Additionally, we performed a similar analysis adjusting for participants, yielding the same conclusion.

#### 2.8.4. HIV Half-Genome Amplification Efficiency

To compare proviral amplification efficiency across MDA methods, the total number of HIV 5′ and 3′ half genomes were tallied across the five participants. Amplification efficiency was calculated by dividing the number of amplified half genomes by the total number of integration sites per MDA method. The 95 CI of the population was also computed.

#### 2.8.5. Cost Comparison

The hands-on time and costs of reagents and plasticware to derive HIV integration sites and to sequence proviral sequences were compared between the HIV MDA and for the REPLI-g MDA.

## 3. Results

The “HIV-MDA” assay was designed to (1) improve the frequency at which the HIV-1 integration sites and the associated proviruses are amplified, by inclusion of 31 primers that anneal to conserved regions of HIV-1, and (2) to reduce the cost of generating data by use of reagents prepared in the laboratory compared to a purchased kit. Primers were designed to anneal to most sequences with alignments that contained at least 100 HIV-1 subtype B sequences (Los Alamos HIV database), with primer lengths adjusted for a T_m_ < 30 °C. Forward primers were designed to amplify the 5′ half of the genome through the 3′ proviral integration site (**IS**), and the reverse primers from the 3′ half through the 5′ IS, using a multiple displacement amplification (**MDA**)-based approach that favors the amplification of long templates. Following optimization of assay conditions, cells collected via leukapheresis from five individuals enrolled in either the PIC or STAR cohorts were amplified by both the HIV-MDA and REPLI-g MDA methods for comparison of yields from the MDA, ISLA, and proviral amplification.

### 3.1. Comparison of the Efficiencies of the Two MDA Methods for Sequencing HIV Integration Sites

**Figure 2:**
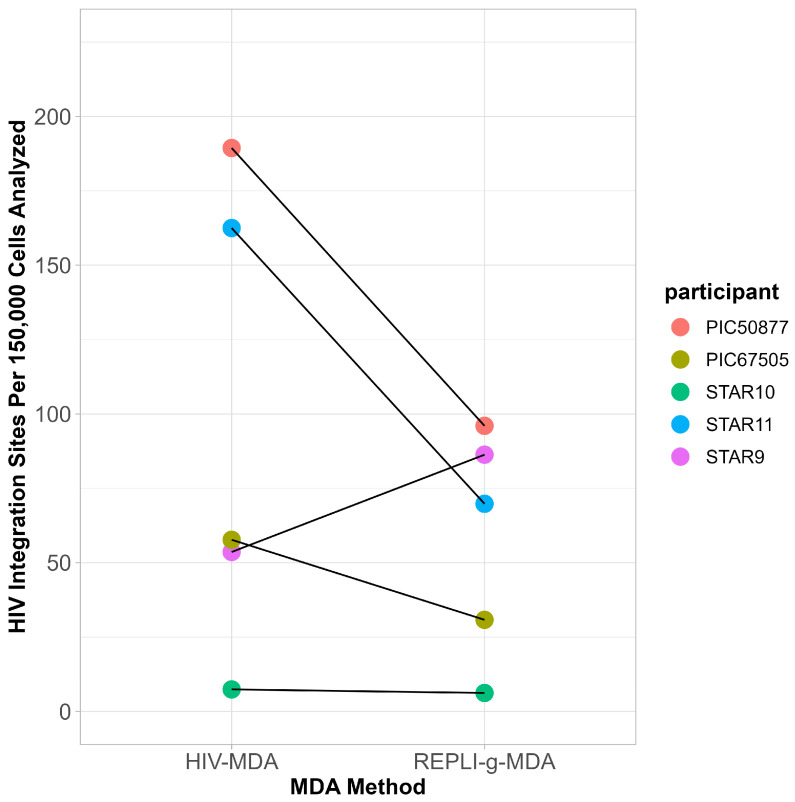
**Comparative yield of HIV integration sites derived by two MDA methods.** The total yield of HIV integration sites (**IS**) generated by HIV-MDA and by the REPLI-g Single Cell kit from leukapheresis specimens from all participants are plotted, expressed as HIV integrations per 150,000 cells.

A comparison of the frequency of HIV IS derived by ISLA from DNA amplified by the two MDA methods found that the HIV-MDA generated relatively more HIV IS compared to the REPLI-g MDA in four of five participants; however, the overall difference between the two MDA methods was not statistically significant (*t*-test, *p* = 0.22) (Figure 2, Table 1).

### 3.2. Comparison of Sampling of the HIV IS Reservoir by the Two MDA Methods

**Figure 3:**
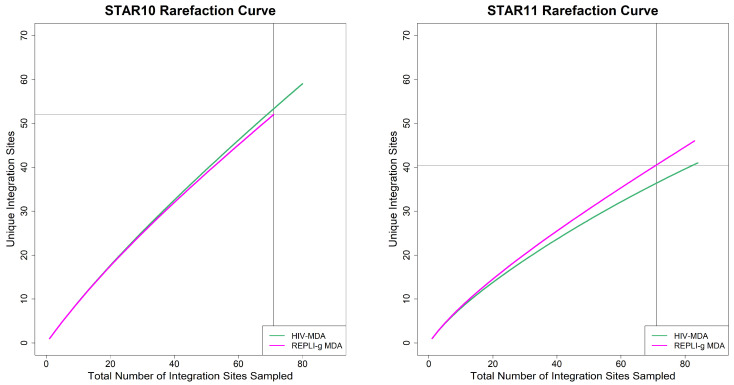
**Analyses of unique HIV integration site sampling across MDA methods.** Rarefaction curves depict the number of unique HIV integration sites (Y axis) detected by repeated sampling of increasing numbers of integration sites (X axis) across each MDA method. Graphs for STAR10 and STAR11 show that despite unequal IS sampling depth, random samples of increasing numbers of IS derived by HIV-MDA and by RELPI-g MDA for STAR10 (**left**) and STAR11 (**right**) detect unique HIV IS at similar rates within these two participants.

We next evaluated whether both methods capture the diversity of the HIV IS reservoir in a similar manner. Rarefaction curve analysis, conducted by tallying the number of HIV IS integrated at unique sites in the human genome from progressively larger random samples of HIV IS, found that HIV-MDA and REPLI-g MDA sample the reservoir similarly (Figure 3 and Figure S1). The steep slopes indicate that the persisting HIV IS are diverse in STAR10 and STAR11 (Figure 3) as well as in the three additional participants (Figure S1). While one can appreciate that the slope of STAR11 flattens slightly, the observation that the slopes (Table S4) do not approach zero (level off) for any of the five participants indicates that, while we generated a relatively large number of HIV IS by both MDA methods, particularly for STAR10 (*n* = 80 and 71) and STAR11 (*n* = 84 and 83), sampling appears to have captured a minor subset of each participants’ HIV IS, making extrapolation of the total number of unique HIV IS in these individuals unreliable (Figure S1).

### 3.3. Reproducibility in the Detection of Clonal HIV IS

**Figure 4:**
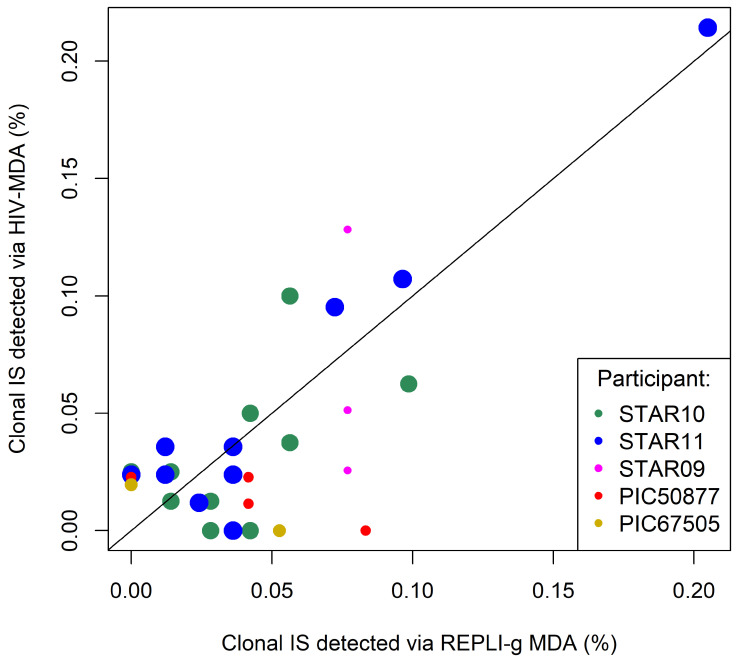
**Comparison of clonal HIV integration sites detected by HIV-MDA and REPLI-g MDA**. Fractions of clonal HIV IS detected by the HIV-MDA and REPLI-g MDA are plotted for the five participants. Each participant’s clones are shown by a single color. Symbol sizes reflect the frequency of each clone’s IS among all IS derived from the participant. The position of the symbol reflects the concentration of the clone within the IS generated for each participant. The diagonal line, which represents theoretical perfect agreement, is added to aid visual interpretation of relative clone concentrations by the two assays.

To assess the quantitative reproducibility of HIV IS generated by the two MDA methods, the detection of clonal HIV IS (defined as HIV IS detected ≥ 2 times in a participant across the two methods) was compared for STAR10 and STAR11; these participants were chosen because each had a substantial number of IS derived by both methods. Linear regression of the clones derived from the two MDA products did not detect significant differences in the quantification of clonal HIV IS (*p* > 0.05 for both samples, see Methods section for details). Additionally, visual evaluation of the combined data over all participants indicates overall clone quantification agreement for both methods (Figure 4).

Given that clonal HIV IS were missed occasionally by one of the two MDA methods, we assessed whether discrepancies between the detection of HIV IS by the two methods could be explained by random sampling effects (Table S3) (see Methods section for more details). Combining clonal IS data failed to reject the hypothesis that the differences observed were due to random sampling effects (*p* = 0.23).

### 3.4. Comparison of HIV Proviral Amplification from HIV-MDA and REPLI-g MDA

To compare the efficiency of HIV proviral DNA amplification from the products of the two MDA methods, amplification of 5′ and 3′ half genomes was performed from MDAs that yielded an HIV IS by ISLA (summarized in Table 2; data from individual participants in Table S5A,B). The yield of amplicons and sequences of 5′ half genomes were tallied if amplicons were generated from reactions using either or both forward primers that targeted potentially intact proviruses or proviruses with deletions in the Major Splice Donor/psi packaging signal region near the 5′ end of the genome. There was an uneven number of primary cells sampled from several participants by the two MDA methods; therefore, a normalized yield of proviral sequences is shown for individuals, calculated by dividing the number of amplified 5′ or 3′ half genomes or near-full-length genome by the total number of integration sites captured for that participant. The efficiency of amplification and generation of HIV 5′ half genomes using two forward primer sets for the HIV-MDA was 25% (100/393) vs. 8% (19/229) for the REPLI-g MDA. The generation of 3′ half genome using one forward primer set also yielded more sequences by HIV-MDA 42% (166/393) vs. REPLI-g 24% (54/229) (Table 2). Similarly, NFLGs assembled from 5′ and 3′ half genomes were derived more frequently from the HIV-MDA, 20% (78/393), compared to REPLI-g MDA, 5.6% (13/229). Alignment of assembled NFLGs showed more major deletions in the 5′ end of proviruses compared to the 3′ end (Figure S2). Identical proviruses were amplified from clonal integration sites by both MDA methods, further validating the HIV-MDA’s ability to accurately amplify proviral genomes (Figure S2).

Assessment of whether the primer binding sites used for half-genome amplification compromised the success of sequencing proviruses, and specifically if the internal half-genome primer binding sites precluded amplification, was performed by NFLG amplification on a subset of MDA wells (*n* = 46). This included wells that yielded HIV IS, including those that yielded none or combinations of 5′ and 3′ half genomes (i.e., 5′−/3′−; 5′−/3′+; 5′+/3′−; and 5′+/3′+) (Table 3). Two different NFLG primer sets (Table S2; two sets of four primers, NFLG or NFLG-alt, lower section of table) were used on the 46 HIV-MDA and REPLI-g MDA wells from a single participant (STAR11). Amplification using these primer sets yielded NFL proviral genomes from 5/46 (11%) of IS-positive and either 5′- and/or 3′-positive HIV-MDA wells vs. 2/46 (4.3%) from the IS-positive and 5′- and 3′-negative REPLI-g wells. The five NFLGs amplified from the HIV-MDA came from 5′+/3′+ wells and previously had NFLG assembled from their half genomes. The two NFLGs amplified from the REPLI-g MDA were both from 5′−/3′− half-genome amplifications and both contained large internal deletions spanning HXB2 1420–9343 or 654–6102; hence, neither the 5′ or 3′ half genomes could have been amplified from these proviruses.

### 3.5. Comparison of Costs to Generate HIV IS and Proviral Sequences by HIV-MDA Versus REPLI-g MDA

The cost of the reagents to perform the two MDA assays in a volume of 40 µL from single HIV-infected cells were compared (Table 4). The cost of reagents for the HIV-MDA reaction and per HIV IS was 13.6-fold lower than for REPLI-g MDA, while the technical hands-on time was the same for both methods. Given that we were only able to amplify and assemble half genomes into near full-length proviral genomes from 20% of the HIV-MDA and 6% of the REPLI-g wells, the cost of the MDA to generate proviral sequences was 46-fold lower for HIV-MDA.

## 4. Discussion

We developed a novel, lower-cost HIV-specific multiple-displacement assay, the HIV-MDA, to amplify HIV-1 genomes in preparation to sequence both the provirus and linked integration site. When compared to a frequently used commercial WGA kit [9,12,14], our HIV-MDA generated greater numbers of HIV IS from most participants and more proviral sequences per infected cell. The two assays generated HIV IS libraries with similar diversities, thus without evidence of primer bias. Importantly, the cost of the HIV-MDA was substantially lower per HIV IS generated, allowing available funds to sample a greater number of HIV IS and proviruses in individuals’ HIV-1 reservoirs.

Our observation that the HIV-MDA compared favorably to REPLI-g MDA suggests that adding the HIV-specific MDA primers alongside random hexamers enhances the amplification of proviral DNA and associated HIV IS relative to use of random hexamers alone. The improvement in amplification of HIV may be because random hexamers amplify virtually all DNA sequences, which, given the low frequency of HIV-infected cells (~1:1000 CD4+ cells), can result in disproportionate amplification of the much more abundant host genomic DNA and reduce effective amplification of viral templates. Targeted amplification of the provirus and associated HIV IS through the addition of HIV-specific primers minimizes the amplification of off-target human DNA that can inhibit downstream PCR in ISLA and proviral amplification [15,26].

The “depth” of sampling the HIV IS reservoir in PBMC was compared between the two MDA methods by detection of unique HIV IS, which was also used to assess primer bias by the HIV-specific primers for amplification of certain HIV IS. While the total number of HIV IS derived from the participants differed, the rarefaction curves of the two MDA assays increased at similar rates in all five participants, suggesting that the HIV-specific primers had minimal, if any, biased amplification. Primer bias was also investigated by a comparison of the two methods for the detection of clonal HIV IS. A linear regression analysis revealed no statistically significant differences between the two MDA methods, with generally similar detection of specific clones and their frequencies across methods and participants. Thus, the use of the wide array of HIV-specific primers we developed appeared to broadly amplify the proviruses in these participants with HIV-1 subtype B infections.

Also notable in the rarefaction analyses was that, with increased sampling of HIV IS, the curves generated from the HIV IS of the five participants did not plateau. This indicates that additional HIV IS are required to ensure reproducible sampling of their HIV reservoirs. If our participants’ HIV IS reservoirs are typical, reproducible sampling of HIV IS and proviral reservoirs may require testing of exceedingly large blood volumes from people with long-term suppression of HIV-1 replication by ART.

Our observation that the HIV-MDA amplified more proviral half genomes (5′ and 3′) compared to REPLI-g MDA again suggests that the HIV-specific primer pools improved the amplification of proviral DNA. However, the amplification of half genomes was not balanced. More 3′ than 5′ half-genome PCR products were successfully amplified from both MDA methods across all five participants we studied for unclear reasons. This imbalance occurred despite the inclusion of forward primers designed to anneal upstream from the multiple splice donor (MSD) and packaging signal site (Ψ), which is known to frequently harbor deletions in ART-suppressed individuals [12,27,28]. To evaluate whether reduced 5′ half-genome amplification was due to internal deletions or mutations at the reverse primer binding site, NFLG amplification was performed. While this experiment was limited to a subset of MDA reactions from a single participant, it did not identify primer mismatches or deletions as the cause; however, we cannot rule out primer biases as a contributing factor. Future approaches incorporating participant-specific primer design, such as sequencing the 5′ LTR, would allow tailoring of primers to specific participants; however, this was not performed for the participants we studied. Given that we only studied five individuals, our findings of unequal 5′ half-genome amplification may not be broadly applicable.

The reduced recovery of 5′ half genomes precluded assembly of NFLGs and evaluation of intact genomes that could potentially rekindle spreading infection if ART is stopped [1,7,29,30,31]. Given the high frequency, >90%, of defective proviral sequences [1,16,17], it is not unexpected that, among our relatively few assembled NFLG, we did not observe intact proviruses. Nonetheless, our results show that the HIV-MDA coupled with long-read sequencing provides a useful approach to confront the challenge of quantitating the infectious viral reservoir.

Others have developed in-house MDA-based methods for sequencing of HIV IS and linked provirus. These include the MDA-SGS [13], which uses random hexamers in the MDA and the IPSA [15], which uses human motif-specific MDA primers. To our knowledge, this study is the first direct comparison of an in-house developed MDA to workflows that use commercial WGA kits for this application.

Limitations of our HIV-MDA include that current HIV-specific primers are designed, optimized and validated only for HIV-1 subtype B. Additionally, our comparison was limited to five individuals, including one, STAR9, with relatively few viable cells available from leukapheresis. We also did not directly compare long-read sequencing with short-read approaches (e.g., Illumina). However, our use of long-read sequencing (i.e., the PacBio platform) allowed us to assemble proviral genomes using reads representing the entire half- and whole-genome amplicons to identify intact vs. deleted forms, whereas short-read methodologies require de novo assembly, which is more labor-intensive [30,32].

A major difference between the two MDA methods we compared was the cost of the assays’ reagents. One HIV-MDA reaction costs less than 1/13th the price of one REPLI-g reaction. Additional cost savings were realized by the relative greater efficiency of the assay for the majority of study participants. Currently, many labs that use the REPLI-g kits to generate HIV IS and sequence associated proviruses (i.e., MIPseq [9]) reduce the volume of the assay to make the assay more affordable, including MDA reactions of 10 µL (versus recommended 50 µL). Even with this change, the per-reaction cost of REPLI-g remains approximately three-fold higher than that of HIV-MDA.

Our novel HIV-MDA compared to a commercial MDA method amplifies more HIV IS and HIV proviruses in most and an equivalent amount in some at a substantially lower cost. A less costly MDA should allow characterization of HIV reservoirs to greater depth and facilitate studies of larger populations.

## Figures and Tables

**Table 1:** Comparative yield of HIV integration sites by two MDA methods. The number and type of cells analyzed, the HIV DNA load quantified by VODA and yield of HIV IS derived by each MDA method are shown for all participants.

Participant	Cell Type	Estimated HIV DNA Load Per 10^6^ Cells	Number of CD4+ * Cells Analyzed		Number of IS Derived		Number of IS Per 150,000 Cells	
			HIV-MDA	REPLI-g MDA	HIV-MDA	REPLI-g MDA	HIV-MDA	REPLI-g MDA
STAR9	CD4+	832	109,142	22,598	39	13	53.6	86.3
STAR10	PBMC	69.1	1,615,672	1,720,620	80	71	7.4	6.2
STAR11	CD4+ PD1 + LAG3−	602	77,541	178,247	84	83	162.5	69.8
PIC50877	CD4+	5632	69,695	37,497	88	24	189.4	96
PIC67505	CD4+	1484	264,936	184,831	102	38	57.7	30.8
	Total:		2,136,985	2,143,793	393	229		

* includes PBMC from STAR10.

**Table 2:** Summary of proviral amplification results from the two MDA methods.

	MDA Method	
	HIV-MDA	REPLI-g MDA
Number of cells analyzed (*n* = 5 assays)	2,136,985	2,143,793
Number of IS identified	393	229
Number of 5′ half genomes amplified	100 (25%, 95% CI: 21–30%)	19 (8%, 95% CI: 5–13%)
Number of 3′ half genomes amplified	166 (42%, 95% CI: 37–49%)	54 (24%, 95% CI: 18–30%)
Number of assembled NFLG	78 (20%, 95% CI: 16–24%)	13 (5.6%, 95% CI: 3–10%)

**Table 3:** Summary of NFLG amplification on a subset (*n* = 46) of MDA reactions from HIV-MDA or REPLI-g MDA.

MDA Method	Number of MDA Wells Tested	Previously 5′ Half-Genome Positive	Previously 3′ Half-Genome Positive	Previously Assembled NFLG (From 5′ + 3′ Half-Genomes)	Amplified NFLG (PCR)
HIV-MDA	46	9/46	19/46	7/46	5/46
REPLI-g MDA	46	0/46	8/46	0/46	2/46

**Table 4:** Comparison of reagent costs for HIV-MDA vs. REPLI-g MDA used for generation of IS and proviral sequences.

MDA Method	Cost Per Reaction	Cost Per 96-Well Plate	Cost Per HIV Integration Site	Cost Per Assembled Near Full-Length Provirus Amplified
HIV-MDA	$1.54	$147.84	$4.62	$23
REPLI-g MDA	$20.95	$2011.20	$62.85	$1048

## Data Availability

HIV integration site data can be found attached (integrationSites_supplemental.xlsx). Proviral half genomes and assembled NFLG sequences can be found attached (Proviral_Sequences.xlsx) and on GenBank with accession codes PX940098–PX940529.

## Supplementary Materials

The following supporting information can be downloaded at: https://www.mdpi.com/article/10.3390/v18030311/s1, Table S1: HIV-MDA forward and reverse primers with binding positions mapped to HIV-1 HXB2; Table S2: Primers used for proviral half-genome or near full-length genome (NFLG) amplification; Table S3: Overdispersion analysis of clonal integration sites to determine if observed differences between both MDA methods are significant or can be attributed to random sampling effects; Figure S1: Unique HIV integration sites detected in repeated random samples of integration sites derived by both MDA methods; Table S4: Rarefaction Curve Slopes; Table S5A: Comparison of 5′ proviral sequencing yield and efficiency by participant; Table S5B: Comparison of 3′ proviral sequencing yield and efficiency by participant; Figure S2: Alignment of Assembled Near Full-Length Genomes (NFLG) derived from both MDA methods; File S1: Integration Sites; File S2: Proviral Sequences.
