# Supplementary material for: Development and Evaluation of a Novel Relatively Low-Cost Method to Derive HIV-1 Integration Sites and Proviral Sequences
Source: Viruses. 2026 Mar 2;18(3):311. doi: 10.3390/v18030311 (PMC13030101; doi:10.3390/v18030311)
Supplement: Supplementary file 1 [file viruses-18-00311-s001.zip › Supplemental_MDAmethods.docx]

Supplementary Materials:

Development and evaluation of a novel relatively low-cost method to derive HIV integration sites and proviral sequences

Samantha R. Hardy ^1^, Sheila Styrchak ^2^, Tim De Meyer ^3^, Laurens Lambrechts ^4^, Tine Struyve ^5^, Basiel Cole ^6^, Liesbet Termote ^7^, Sherry McLaughlin ^8,^ James I. Mullins ^9^, Linos Vandekerckhove ^10^, and Lisa M. ­­­Frenkel ^11^*

**Table S1:** HIV-MDA forward and reverse primers with binding positions mapped to HIV-1 HXB2

| **HIV-MDA Primers** | | | |
| --- | --- | --- | --- |
| **Primer:** | **Sequence** | **HXB2 position:** | **Primer Pool** |
| MDA.F10s | CACCAAATG*A*A | 2050-2060 | Forward |
| MDA.F11s | CTGTACCA*G*T | 2569-2578 | Forward |
| MDA.F12s | TCCATCCT*G*A | 3250-3259 | Forward |
| MDA.F13s | AACAAATCAG*A*A | 3965-3976 | Forward |
| MDA.F14s | ATTCCCTAC*A*A | 4650-4660 | Forward |
| MDA.F15s | CATAGAATG*G*A | 5298-5308 | Forward |
| MDA.F16s | GCAGGAAG*A*A | 5973-5982 | Forward |
| MDA.F2s | GAGGCTAG*A*A | 772-781 | Forward |
| MDA.F3s | GGAGAGAG*A*T | 782-791 | Forward |
| MDA.F4s | GTATGGGC*A*A | 892-901 | Forward |
| MDA.F5s | CAGAAGAACT*T*A | 1004-1015 | Forward |
| MDA.F6s | GAAGCTTTA*G*A | 1084-1094 | Forward |
| MDA.F7s | AACAAAAGTAA*G*A | 1114-1126 | Forward |
| MDA.F8s | TGGGTAAAA*G*T | 1252-1262 | Forward |
| MDA.F9s | TACCCATG*T*T | 1295-1304 | Forward |
| MDA.R10s | TTACTGCTT*T*G | 6041-6049 | Reverse |
| MDA.R11s | TTCTGAAAAA*C*A | 5769-5779 | Reverse |
| MDA.R12s | GTACTGCT*G*T | 4749-4758 | Reverse |
| MDA.R13s | GTCTGTTACT*A*T | 4032-4043 | Reverse |
| MDA.R1s | TGACTGGA*A*A | 8998-9007 | Reverse |
| MDA.R2s | AAGTCTCTC*A*A | 8535-8545 | Reverse |
| MDA.R3s | AACCCAAG*G*A | 7780-7789 | Reverse |
| MDA.R4s | CCACTCTT*C*T | 7731-7740 | Reverse |
| MDA.R5s | CTAATGGTTC*A*A | 7696-7707 | Reverse |
| MDA.R6s | CAGGTCTG*A*A | 7626-7635 | Reverse |
| MDA.R7s | CTCCACAATT*A*A | 7351-7362 | Reverse |
| MDA.R8s | AGTTGAGTT*G*A | 6990-7000 | Reverse |
| MDA.R9s | TGTTCTACC*A*T | 6522-6532 | Reverse |
| MDA.R26 | CACCATCTC*T*T | 7589-7599 | Reverse |
| MDA.R25 | CTACTTTATATTTA*T*A | 7674-7689 | Reverse |
| MDA.R24 | TCTTTTTTCT*C*T | 7746-7757 | Reverse |
| MDA.R23 | AAGAACCC*A*A | 7783-7792 | Reverse |
| MDA.R22 | CATAGTGC*T*T | 7805-7814 | Reverse |
| MDA.R21 | TACCAGACAA*T*A | 7855-7866 | Reverse |
| MDA.R20 | CAACCCCA*A*A | 8008-8017 | Reverse |
| MDA.R19 | TTACTCCAAC*T*A | 8060-8071 | Reverse |
| MDA.R18 | AACCAATTC*C*A | 8232-8242 | Reverse |
| MDA.R17 | CTATCATTATGAA*T*A | 8275-8289 | Reverse |
| MDA.R16 | CTTCGATTC*C*T | 8411-8422 | Reverse |
| MDA.R15 | TCAAGAGTAA*G*T | 8542-8553 | Reverse |
| MDA.R14 | CCCTATCT*G*T | 8700-8709 | Reverse |
| Random Hexamer | NNNN*N*N |  |  |

*Indicates phosphorothioate bonds

**Table S2**. Primers used for proviral half-genome or near full-length genome (**NFLG**) amplification

| **Primer Name:** | **Sequence (5'-3')** | **HXB2 Position** | **PCR round** | **Amplicon Type** |
| --- | --- | --- | --- | --- |
| F544 | TTAAGCCTCAATAAAGCTTGCCTTGAG | 518 - 544; 9603 - 9629 | forward, 1st round | 5’Half-genome |
| R5968 | TGTCTYCKCTTCTTCCTGCCATAG | 5991 - 5968 | Reverse, 1st round | 5’Half-genome |
| PB-F_F581 | **TCGTCGGCAGCGTC**GTGTGCCCGTCTGTTGTGTGACTC | 558 - 581; 9643 - 9666 | forward, 2nd round, with **PacBio adapter** | 5’Half-genome |
| PB-R_R5783 | **GTCTCGTGGGCTCGG**AATGCCTATTCTGCTATGTYGACACC | 5808 - 5783 | Reverse, 2nd round, with **PacBio adapter** | 5’Half-genome |
| F423 | GGAGTGGCGARCCCTCAGATGCT | 401-423; 9486-9508 | Alt- forward, 1st round | 5’Half-genome |
| PB-F_F469 | **TCGTCGGCAGCGTC**CYTGTACTGGGTCTCTCTRGTTAG | 446-469; 9531-9554 | Alt- forward, 2nd round, with **PacBio adapter** | 5’Half-genome |
| F5066alt1 | TATGGAAAACAGATGGCAGGTGMTGRT | 5040 - 5066 | forward, 1st round | 3’Half-genome |
| R9665 | GTCTGAGGGATCTCTAGWTACCAGA | 9689 - 9665; 604 - 580 | Reverse, 1st round | 3’Half-genome |
| PB-F_F5088alt1 | **TCGTCGGCAGCGTC**GATTGTGTGGCARGTAGACAGRATG | 5064 - 5088 | forward, 2nd round, with **PacBio adapter** | 3’Half-genome |
| PB-R_R9602 | **GTCTCGTGGGCTCGG**CAAGGCAAGCTTTATTGAGGCTTAAS | 9627 - 9602; 542 - 517 | Reverse, 2nd round, with **PacBio adapter** | 3’Half-genome |
| 638F | GCGCCCGAACAGGGACYTGAAARCGAAAG | 638-666 | forward, 1st round, NFL-alt | NFLG-alt |
| BLOuterR | TGAGGGATCTCTAGTTACCAGAGTC | 9662-9686; 577-601 | Reverse, 1st round, NFL-alt | NFLG-alt |
| PB-263F | **TCGTCGGCAGCGTC**GACCTGAAAGCGAAAGGGAAAC | 651-672 | forward, 2nd round, with **PacBio adapter,** NFL-alt | NFLG-alt |
| PB-280R | **GTCTCGTGGGCTCGG**CTAGTTACCAGAGTCACACAACAGACG | 9650-9676; 565-591 | Reverse, 2nd round, with **PacBio adapter,** NFL-alt | NFLG-alt |
| F544 | TTAAGCCTCAATAAAGCTTGCCTTGAG | 518 - 544; 9603 - 9629 | forward, 1st round | NFLG |
| R9665 | GTCTGAGGGATCTCTAGWTACCAGA | 9689 - 9665; 604 - 580 | Reverse, 1st round | NFLG |
| PB-F_F581 | **TCGTCGGCAGCGTC**GTGTGCCCGTCTGTTGTGTGACTC | 558 - 581; 9643 - 9666 | forward, 2nd round, with **PacBio adapter** | NFLG |
| PB-R_R9602 | **GTCTCGTGGGCTCGG**CAAGGCAAGCTTTATTGAGGCTTAAS | 9627 - 9602; 542 - 517 | Reverse, 2nd round, with **PacBio adapter** | NFLG |

| **Table S3.** Overdispersion analysis of clonal integration sites to determine if observed differences between both MDA methods are significant or can be attributed to random sampling effects | | | | |
| --- | --- | --- | --- | --- |
| **Participant** | **One Sample T-test** | | **Exact Binomial Test** | |
|  | **p-value** | **95 CI** | **p-value** | **95 CI** |
| **STAR10** | 0.9066 | [-1.36, 1.22] | 0.5811 | [0.139, 0.684] |
| **STAR11** | 0.1483 | [-4.76, 0.797] | 0.6072 | [0.322, 0.836] |
| **STAR9** | 0.5534 | [-7.64, 10.64] | 1 | [0.094, 0.99] |
| **PIC50877** | 0.0055 | [0.46, 1.73] | 0.125 | [0.0036, 0.579] |
| **PIC67505** | *insufficient data | * insufficient data | * insufficient data | * insufficient data |

**
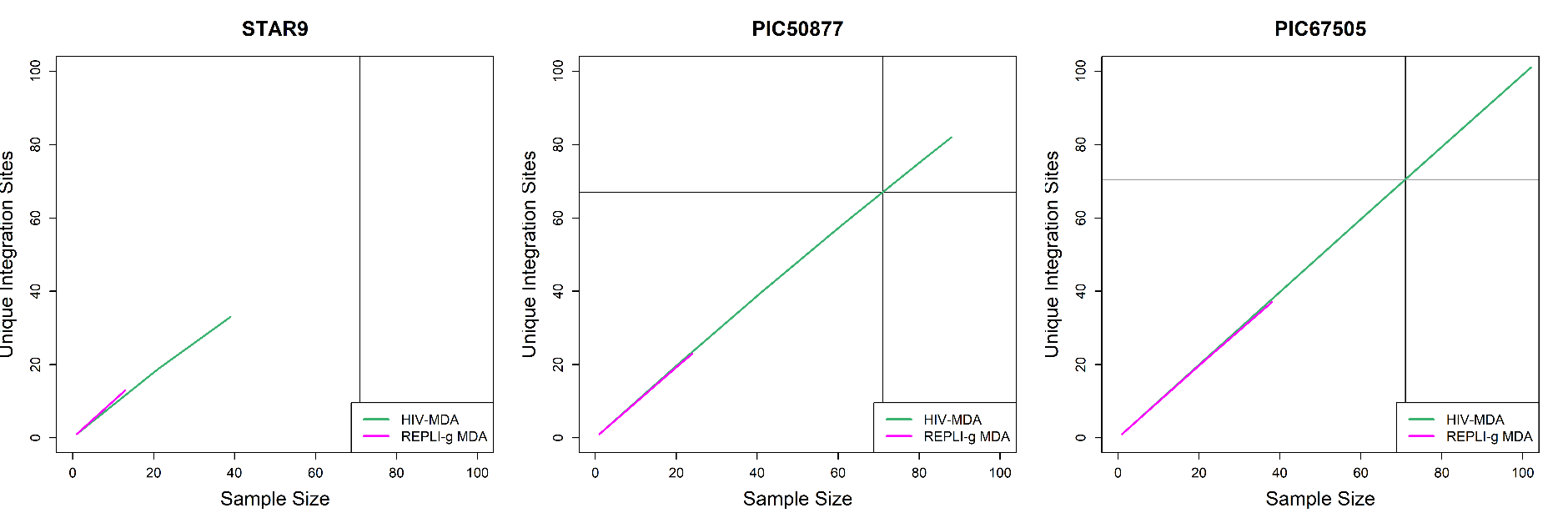
**

**Figure S1**. **Unique HIV integration sites detected in repeated random samples of integration sites derived by both MDA methods.** Rarefaction curves depict the number of unique HIV integration sites (Y axis) detected in STAR 9 (left), PIC 50877 (center) and PIC 67505 (right) by repeated sampling of increasing numbers of integration sites (X axis).

| **Table S4.** Rarefaction Curve Slopes | | |
| --- | --- | --- |
| **Participant** | **HIV-MDA** | **REPLI-g MDA** |
| STAR10 | 0.71 | 0.70 |
| STAR11 | 0.45 | 0.51 |
| STAR9 | 0.86 | N/A |
| PIC50877 | 0.96 | N/A |
| PIC67505 | 0.99 | 0.95 |

| **Table S5A.** Comparison of 5’ proviral sequencing yield and efficiency by participant | | | | |
| --- | --- | --- | --- | --- |
| **Participant** | **Total number of amplified 5’ HIV half genomes** | | **5’ half genome amplification efficiency from ISLA+ well** | |
|  | **HIV-MDA** | **REPLI-g MDA** | **HIV-MDA** | **REPLI-g MDA** |
| STAR9 | 2 | 0 | 5% | 0% |
| STAR10 | 22 | 15 | 28% | 21% |
| STAR11 | 24 | 0 | 29% | 0% |
| PIC50877 | 22 | 0 | 25% | 0% |
| PIC67505 | 30 | 4 | 29% | 11% |
| **Total:** | **100** | **19** | **25%** | **8%** |

| **Table S5B.** Comparison of 3’ proviral sequencing yield and efficiency by participant | | | | |
| --- | --- | --- | --- | --- |
| **Participant** | **Total number of amplified 3’ HIV half genomes** | | **3’ half genome amplification efficiency from ISLA+ well** | |
|  | **HIV-MDA** | **REPLI-g MDA** | **HIV-MDA** | **REPLI-g MDA** |
| STAR9 | 5 | 0 | 13% | 0% |
| STAR10 | 40 | 29 | 50% | 41% |
| STAR11 | 37 | 13 | 44% | 16% |
| PIC50877 | 41 | 1 | 47% | 4% |
| PIC67505 | 43 | 11 | 42% | 29% |
| **Total:** | **166** | **54** | **42%** | **24%** |

**
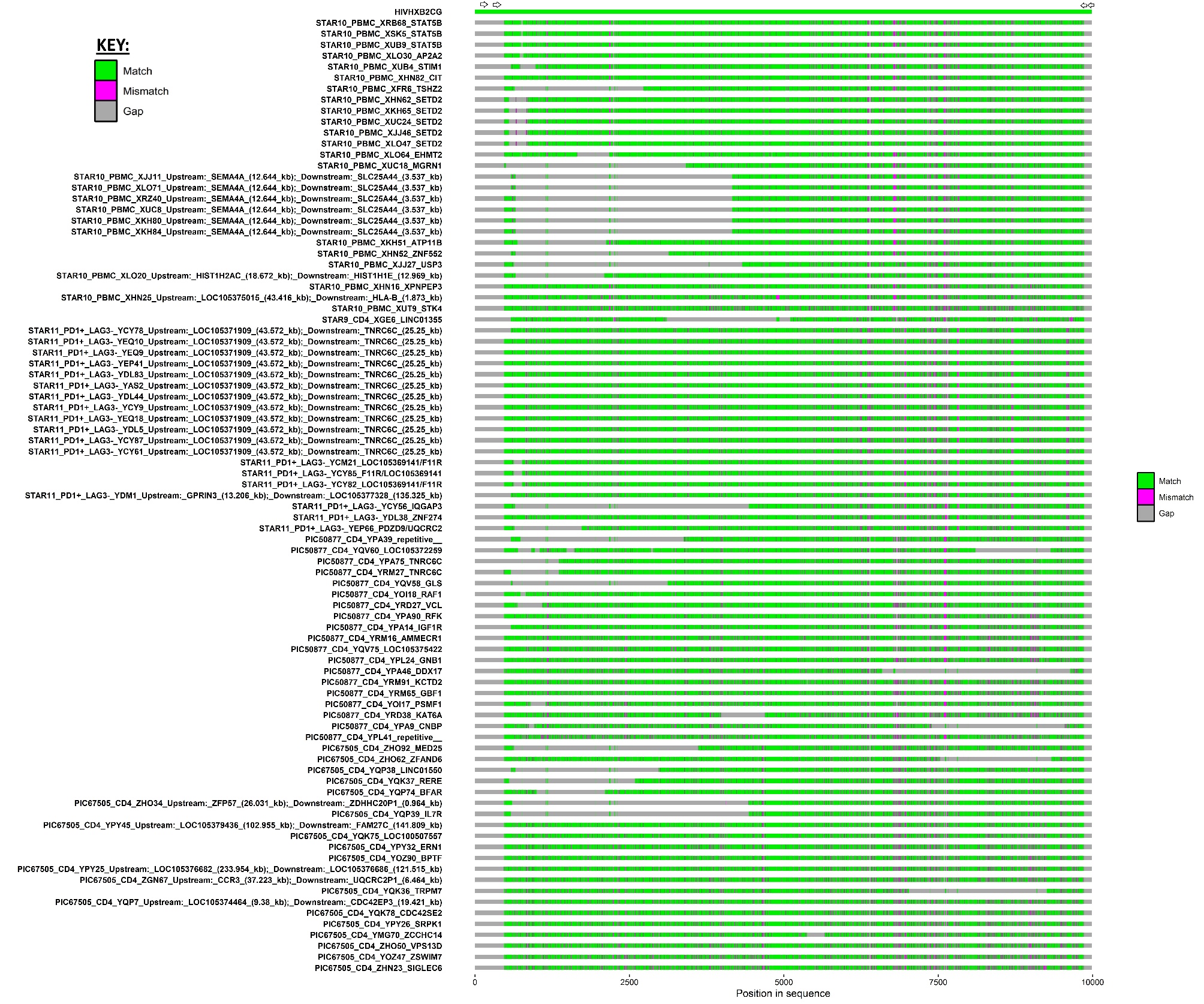
Figure S2. Alignment of Assembled Near-Full Length Genomes (NFLG) derived from both MDA methods.** 5’ and 3’ half genomes from all participants with identical sequences in the overlapping region are aligned to HXB2. Text on left indicates participant ID, cell type assayed, reaction code and name of gene in which provirus is integrated.
